# Carrying on life at home or moving to a nursing home: frail older people’s experiences of at-homeness

**DOI:** 10.1080/17482631.2022.2082125

**Published:** 2022-05-29

**Authors:** Bente Egge Søvde, Anne Marie Sandvoll, Eli Natvik, Jorunn Drageset

**Affiliations:** a Department of Health and Caring Sciences, Western Norway University of Applied Sciences, Førde, NORWAY; b Department of Global Public Health and Primary Care, University of Bergen Faculty of Medicine and Dentistry, Bergen, Norway; c Department of Global Public Health and Primary Care, University of Bergen Faculty of Medicine, BERGEN, Norway; d Department of Health and Social Sciences, Western Norway University of Applied Sciences, Campus Bergen, Bergen, Norway

**Keywords:** Frail older people, phenomenology, at-homeness, in-depth interview

## Abstract

**Aims and objectives:**

The aim was to explore frail older people’s lived experiences of managing life at home on the verge of moving to a nursing home.

**Background:**

As people age, their reserve capacity decreases, increasingthe risk of morbidity and frailty.. The experience of frailty extends beyond declining health and physical well-being and encompasses various dimensions, including familiarity with both the place and the people around.

**Design:**

A phenomenological study.

**Methods:**

We interviewed ten frail people aged 72–90 years in-depth in their homes. We used phenomenological hermeneutical analysis inspired by van Manen and followed the COREQ checklist.

**Results:**

We identified three main themes: (1) being home with cherished people and possessions, (2) giving the nursing home a go and (3) attuning to the natural rhythms.

**Conclusions:**

Our study gives insight into the lived experiences with frailty related to at-homeness. The experience of being lost in transition represents a uniquely significant experience for frail older people, foregrounding existential issues and carrying the potential of at-homeness.

**Relevance to practice:**

To unleash frail older people’s potential for at-homeness, health professionals mustmeet the needs of frail older people individually. Going beyond signs and symptoms to reveal people’s concrete everyday experiences is crucial to understanding frailty .

## Introduction

The rapid growth in the ageing population in terms of both number and longevity has drawn global attention to the needs of older people, especially those who are frail (WHO, 2017). Well-being is a subjective experience that can prevail even in the presence of ill health when balancing older people’s resources and challenges (Dodge et al., 2012; Eriksson & Eriksson, 2018). Frailty, defined as the presence of several interacting medical and functional problems associated with low well-being, makes frail older people more vulnerable to adverse outcomes (Clegg et al., 2013; Fried et al., 2001; Kojima et al., 2019). As a compelling global public health issue, frailty affects individuals, families, communities and society (Lekan et al., 2021). Decreasing well-being and increasing levels of frailty might lead to impaired quality of life and loneliness and apply to mental and social functioning (Hoogendijk et al., 2019; Warmoth, 2016).

Ageing at home has been a trend in Scandinavia for several decades (Ministry of Health and Care Services, Norway, 2018; Walker et al., 2015). Still, in many Western countries older people move from their ordinary homes to institutional care for the final period of their lives (Saarnio et al., 2018, 2017; Statistics Norway, 2019). In Norway, more than 90% of places in nursing homes are public, and public services commonly offer a short-term stay of 4–6 weeks for older people with deteriorating health (Statistics Norway, 2019).

Home is regarded as the place where people recognize themselves, a place where one is known and seen by others, manages oneself, is close to significant others, experiences love and friendship, and is safe (Saarnio et al., 2018). A home is a place where they are familiar with the surrounding place and people, feeling safe, connected and centred (Öhlén et al., 2014).

In Norway, people have continually shaped their homes, linked to daily, weekly, and annual cycles with seasonal and ritual aspects (Pasveer et al., 2020). According to Hilli and Eriksson (2019), the source of vitality lies in the home, where one can show one’s true self and innermost feelings.

Strategies for enabling a feeling of at-homeness are consistent with a personalized approach to promoting health and well-being, such as socializing and maintaining independence (Frost et al., 2017, 2018; Saarnio et al., 2019). Moving away from home makes people particularly aware of the importance of the homeness of their home (Baldursson, 2002). Studies from nursing homes describe that living there is considered a lonely and homeless existence (Österlind et al., 2017; Sjöberg et al., 2019). Moving to a nursing home is life-changing, and reported consequences are loss of autonomy, independence and identity (O’Neill et al., 2020).

Ageing populations all over the world face the challenge of maintaining older people’s well-being (Clegg et al., 2013; Dodge et al., 2012; Fried et al., 2001; Hoogendijk et al., 2019; Kojima et al., 2019; Warmoth, 2016). Previous research has emphasized that a home is a special place where older people have experienced belonging, security and well-being (Öhlén et al., 2014; Pasveer et al., 2020; Saarnio et al., 2018). Health, well-being and at-homeness urgently need to be promoted throughout frail older people’s lives, as many move to nursing homes in the final stages of life, feeling estranged and lonely (Österlind et al., 2017; Sjöberg et al., 2019). The physical changes connected to frailty are subtle and progressive and significantly predict nursing home placement (Clegg et al., 2013;; Lekan et al., 2021; Österlind et al., 2017). Phenomenological studies that examine the negative consequences of frail older people’s experiences of being on the verge of living at home and moving to a nursing home are sparse. New insights into frail older people’s experience of at-homeness are decisive for planning and caring for this group of people. Studies from the first-person perspective can challenge established assumptions and provide valuable insight to formal and informal caregivers and society. The aim was to explore frail older people’s lived experiences of managing life at home on the verge of moving to a nursing home. Our study’s research question was: How do frail older people experience at-homeness?

## Theoretical framework

Phenomenology is a perspective addressing the foundations of knowledge and its development, often guiding methods appropriate for qualitative health research. Lived experience, subjectivity and the lifeworld are at the core (Heidegger, 1962). The lifeworld is the world we live in, the world of experience we take for granted in daily life (Van Manen, 2014). Our inquiry, reflections, and analysis were guided by the approach of Van Manen (2014). Phenomenological concepts are central to our empirical data analysis, such as vulnerability, dependence on others, relationships, mortality and existential loneliness (Heidegger, 1962; Vetlesen, 2009). Svenaeus (2010) progressed a view of health as a “homelike being in the world”. At-homeness might be considered an aspect of well-being despite illness for frail older people. When healthy, we are attuned to our lifeworld, whereas in illness, the natural rhythm of attunement is replaced with a sense of an unhomelike being in the world (Cooney, 2012).

## Methods

We conducted a phenomenological study inspired by the methods of van Manen (Van Manen, 2014) and followed the COREQ checklist. The fundamental phenomenological question is, “How is this experience?”. This question allows us to wonder about the meaning of a particular moment of lived life.

## Participants and recruitment

We conducted a purposive sampling strategy and searched for participants to provide insight into the phenomenon under study (Patton, 2015). Participants older than 65 years with frailty according to the phenotype model of Fried et al. (2001) and a Mini-Mental State Examination score over 18 showing minimal cognitive impairment were recruited with head nurses’ help from two geriatric outpatient clinics. The participants were 72–90 years old. Four lived alone, and six lived with their partner. One withdrew because of deteriorating health. The participants lived at home, and because of their frailty, they have had temporary stays at nursing homes or rehabilitation units.

Table I presents the characteristics of the study participants.

**Table I.** Characteristics of study participants (*n* = 10)

Characteristics	*n* (%)
**Sex**	
Male	3
Female	7
**Age (years)**	
70–74	1
75–79	1
80–84	3
85–89	3
90–94	2
**Help**	
Formal help^a^	10
Informal help^b^	10
**Form of living and living place**	
Own house with a spouse	4
Own house alone	4
Own house with spouse and family	2
**Experience of institutional stay**^c^	
Nursing home	8
No nursing home	2

^a^Health care providers at home health care, nursing homes, hospital wards and outpatient clinics. Regular general practitioner, physiotherapist.

^b^Informal caregivers such as spouses, children, in-laws, siblings, friends or neighbours.

^c^In Norway, brief stays in nursing homes for 4–6 weeks are common.

We interviewed 10 older people in-depth about their experiences living at home with frailty in December 2018–2019. Before data collection, the first author conducted a pilot interview to ensure that the questions’ wording, flow and order were natural and understandable. The first author conducted in-depth face-to-face interviews in the participants’ homes to ensure a familiar environment. The style of the interviews was informal, framed as conversations to bring out natural accounts of the everyday experiences we sought to explore. The interview incorporated semi-structured and unstructured interview elements to minimal rigidity and maximum depth. The interviews aimed to capture a detailed description of the phenomenon of at-homeness from the perspective of frail older people (Brinkmann & Kvale, 2014). The researchers included one PhD student with several years of experience in home health care and three experienced health researchers. The research team had experience in quantitative and qualitative research, phenomenology and ageing. We sought to capture participants’ pre-reflective experiences, which refer to the moment as it was lived rather than how it is theorized, conceptualized or categorized (Merleau-Ponty, 2002). In addition to the verbal conversation, all but two participants voluntarily gave a tour of their homes, where participants identified meaningful objects and spaces for the researcher. The first author wrote reflective notes about the home’s mood, content, setting and, more generally, the impression after each interview. These notes provided contextual information for data analysis.

## Data analysis

The interpretive data analysis was inspired by Van Manen’s (1990) iterative and inductive framework for phenomenological data. The method was to read and rewrite, present preliminary interpretations and questions and highlight important sections to restore the structure of meanings embodied in the human experience represented in the text. The analysis started with all authors reading through the interviews to get an overall impression. Furthermore, we identified and organized themes by collecting excerpts, asking various questions and seeking an understanding of the phenomenon’s essential meanings.

After reading each interview in detail, the first author wrote short reflective notes, supported by reflections from a research diary. These notes helped identify key concepts and topics that were common or immersed and warranted further research. The first author prepared the analysis and discussed the new topics with the group of researchers (JD, AMS and EN). The authors met and regularly discussed during the whole process. We had several rounds to clarify, concretize and abstract the material content of the material and develop themes. The last phase of the data analysis was developing and describing the themes of the phenomenon to obtain more profound knowledge of older people’s experiences during the whole process (Van Manen, 2014). The analysis resulted in three final themes: (1) being home—where one lives with cherished people and possessions, (2) giving the nursing home a go and (3) attuning to the natural rhythms.

## Ethical considerations

All participants gave written and oral consent before interviews were conducted. We presented the study to the Western Norway Regional Committee for Medical and Health Research Ethics, and the Norwegian Centre for Research data approved it (Ref. 61,202). We followed ethical guidelines (“World Medical Association Declaration of Helsinki: Ethical Principles for Medical Research Involving Human Subjects,” 2013).

## Findings

Experiencing institutional life was twofold, with a different form of security and easily accessible help from health workers. Experiencing frailty led to a balancing act where the participants lived on the verge of managing at home and moving to a nursing home. Their lives and homes intertwined with their stories, rituals and memories. The home was a shelter where they could return and feel safe. As the body changed and became frailer, the home was no longer sufficiently safe. A temporary stay in an institution was inevitable. An overall finding was that the participants wanted to regain a feeling of at-homeness in their experience of not being at home. Having a short-term stay at the nursing home reinforced a sense of limited time, that the life they knew and appreciated would soon be over. Existential issues, including its finiteness and meaning, became more apparent. The participants described having no one to talk to about these problems and expressed themselves by opposing moving into a nursing home. Participants knew that staying at home was barely possible since their unpredictable bodies made this more troublesome. Even though we presented the three themes separately below, they were coherent and overlapping.

## Being home—with cherished people and possessions

The first theme comprised participants’ descriptions of grounding and rootedness at home, manifesting the blurred borders between self, others and the world. First, and most importantly, the home entailed shared time and space with significant others. Being at home included locality and landscapes, participants’ gardens, dwellings and views. Cherished possessions enabled and enhanced the feeling of at-homeness and well-being. The participants used their homes and objects, such as family photos, paintings, diplomas, crafts and family treasures, to tell their identities and stories of settlement.

One 90-year-old participant linked feeling alive to living at home, even if frailty made her dependent on her husband. The home had a steep staircase, and she could not go outdoors by herself, but with him, she could. The experience of breathing fresh air in the surrounding nature was vital. Even if she could not walk in the terrain, the couple went for a drive every Sunday to a place with a nice view.

I think I am fine. We are both 90 years old. My husband manages to arrange the garden and everything; he goes shopping and cooks every meal. I get served breakfast every morning, and he prepares the dinner. I hope to continue as it is, with medication and all of that. Moreover, physically and mentally: it is best to be home! (participant 9)

Participants felt at home and complete when with their spouse, experiencing kinship and belonging. In this, they pointed to close family and friends as crucial aspects of their experience of at-homeness. They were aware of their reduced lifespan and reduced capability to manage everyday life. The home was where they spent their lives, where they had built a life, and they fought to be able to stay there. Some expressed reluctance, others resisted, and a few rejected the idea of moving out of their home altogether. The resistance to leaving home was linked to experiencing emptiness and homelessness for oneself and, for some, one’s spouse. They were worried about getting along without each other.

One woman described the loss of both her spouse and her home. She had longed for his closeness and the place they spent together as a family. She and her husband planned to spend their retired life in her hometown. Unfortunately, shortly after they moved, he got severely ill and died.

I have made a big mistake; I moved from the city where I lived for 42 years. I miss my friends; I miss the street. Here, I have no friends left. (participant 2)

She felt alone despite daily phone calls with her children. She missed the togetherness and life she had before, where she knew people, and they knew her. It was her hometown, but she did not feel at home. They had planned for a new beginning that instead became an abrupt ending, and now, she tried her best to start a new life on her own. She visited people at the nursing home, and she dined with a relative at the cafeteria once a week. Nevertheless, she missed feeling connected to the place.

The fear of being left alone was pivotal for the participants. For example, a 72-year-old woman with reduced lung capacity lived with her husband, and she described being restless when away from home, anxious about becoming acutely ill and dying without her loved ones by her side.

I do not want to be away from home. I am afraid that I will not get enough time here … The doctor and my family have asked me if I would like a (temporary) stay at the nursing home. Just thinking about it gives me a stomach ache. Even if I could get help with inhalations and syringes and whatever it may be, it is almost like a nightmare thinking about it. I would feel very lonely, even if I had a hundred people around me. It is like none of it is mine over there. I do not want to be away from home. (participant 1)

She depended on others and received care, but a temporary stay at a nursing home was out of the question. She clearly said that this was something she feared deeply; leaving home was the worst-case scenario for her.

## Giving the nursing home a go

Participants knew they were on the verge of not being able to manage at home, even with help from other people. Recently, frailty had led to a temporary stay at a nursing home, hospital or rehabilitation unit for all of them. The institution’s interior was adapted for patients, making it easier to move around. Participants described a longing to breathe fresh air but found walking outdoors by themselves challenging. Some participants were offered trips outside by healthcare providers, which they appreciated, but some had experienced the opposite. For example, during a three-month rehabilitation stay, one participant’s goal was to walk outside with his leg prosthesis. He trusted healthcare providers to provide him with a walk outside, but it did not happen.

I did not dare to go out either because I had amputated my foot, and there is a steep hill [outside the nursing home]. I feared I would be unable to walk back up the hill by myself. If I had asked someone, they might have assisted me. (participant 7)

The participants, overall, demonstrated being treated and cared for by healthcare providers. Despite this, several experienced the nursing home with long days and without content, saying they sat there, just waiting for the next meal. They experienced emptiness, fearing that this was their fate. They wanted to decide for themselves, such as their morning routine, preparing meals or walking outside a little.

Participants said they felt seen and recognized by most healthcare providers but not by all employees. For example, one participant had difficulty putting on her pantyhose in the morning and decided to wear them overnight. On the night shift, a nurse noticed this.

One of the older nurses told me to take off my pantyhose [laughter]. I thought it was funny, but I had to keep myself from laughing. I took it seriously, of course, when she said it so strictly. After this, healthcare providers came after I had gone to bed, standing behind the headboard and looking down. I am sure they were sent to check on me. Especially a couple of them acted like, “this is how we do it around here”. (participant 3)

Several participants mentioned that they appreciated the conversations with healthcare providers, looking forward to seeing and talking to them. Some of the employees made an extra effort, stopping by to talk about the news or sharing something from their personal lives, which was highly valued. A visit or a phone call from family lit up the participants’ day. In addition, the more extroverted residents started conversations at the dinner table.

It was a bit fulfilling to be there. The healthcare providers were so eager to have me dine with the others. I was myself, talking and laughing. The other residents were pleased when I arrived. Moreover, I felt that it was an excellent place to be. You got all the help you needed and more. However, I felt that my head did not quite fit there, and my body did because it was lousy. (participant 3)

The participant thought that it was encouraging that the other residents appreciated her company and that the employees considered her a resource. She received rehabilitation for her health problem at the nursing home. Nevertheless, she doubted that this was the best place to stay in the future.

Being offered a long-term stay at a nursing home was an eye-opening experience for the participants. They started considering what that could be like for them. From that point, moving into a nursing home became a definite possibility for the near future.

A 90-year-old man had ambiguous experiences in the nursing home, and he described it like this:

Many older people were at death’s door, and it was a reminder that this was the last place you would stay. Nevertheless, there was church service once a week, and some of the residents were interesting to talk with. As a 95-year-old woman – she was a storyteller – told stories from old times. Nevertheless, I was not so fond of the other activities at the nursing home; some clowns had more appropriate performances for children than for older people – no, being away from home, in a hospital or a nursing home. I think I would die within a year. (participant 7)

Participants asked themselves whether they could manage at home or whether it was time to reconcile themselves with life at the nursing home. There were some positive aspects of living at the nursing home, such as getting new acquaintances and being surrounded by caring employees. However, participants said that a long-term stay was a step closer to the end. They described the main issue with staying at the nursing home: the absence of the life they had built and lived at home, the life they knew and highly appreciated. In addition, the at-homeness they felt about their home felt impossible to accomplish because of the institution’s different and alienating rhythm and rules.

## Attuning to the natural rhythms

After returning home from a stay at an institution, everyday life followed a natural and familiar rhythm. The participants existed between movement and stillness, being inside and outdoors, alone and together with others. Participants managed their household, prepared meals, talked with their partners, watched TV, fed the birds and gazed at life outside the window. However, life was changing, and they needed extended help and support from others to manage their natural daily rhythms. Next of kin typically planned for their homecoming by rebuilding their house, extending the bathroom, or facilitating the entrance to avoid stairs. Healthcare providers helped with medication, injections, inhalations, personal hygiene and wound care. Participants received safety alarms, walking aids, specialized chairs and dinner delivered from the nursing home. Even if participants planned to continue at home, life did not always turn out as planned.

We have dinner delivered every day except Sunday. I can make dinner, but I will have to spend all day by the stove. Of course, I miss it a bit, but we have a big house, so the days pass anyway. (participant 8)

Contributing to the daily practicalities of homemaking was pivotal for her experience of being home. Holding on to what she managed, such as preparing potatoes for Sunday dinner, was essential.

After an extended stay at a hospital and nursing home, a woman returned home, describing vitality because of rehabilitation. However, she longed to use her energy planning and perform the family farm’s seasonal activities for generations.

I enjoy working with wood. When spring is here, and my nephew has brought timber from the forest, I sit out in the yard with my old-fashioned saw. I had a great time this summer. I sat outside with the walker. Suddenly a robin came and sat down in front of me. I sat still, and it was lovely! We have deer as well. We have trees above the house. It was the time of the year when the leaves were about to emerge. Then I saw a deer standing on two legs. He stretched to get hold of the freshly sprouted leaf to the edge. And he got it! There was no deer track here when I was away and no birds. This means that the animals notice whether people are at home or not. It was utterly dead here [when I returned from the nursing home]. Now the birds are back. (participant 3)

She enjoyed working outside when she had the energy for it, and she appreciated the calmness and stillness and forced herself to make herself open to sensing her surroundings. When she returned from the nursing home, she noticed that the place seemed empty and abandoned. She experienced adding life to her home and the surrounding nature adding life to her. She expressed her home and herself as intertwined and mutually dependent on each other to bring life to their spot in the world.

Participants experienced the rhythm of nature as predictable and safe, in contrast to their frail bodies and unpredictable future. These rhythms guide the daily activities of all living beings, such as changes in daylight, temperature and weather throughout the day, from season to season, generation to generation. The participants did not take this rhythm for granted, and it was not just a backdrop for their daily activities. Observing and sensing nature seemed to make them rest in their presence. Own decline and mortality were backgrounded. Participants wanted to feel well here and now. In nature, they found moments of peace and tranquillity. The participants expressed this as experiential wisdom, telling them what they needed and perhaps had to let go.

We have talked a bit about what happens afterwards. My husband and I are good at solving global problems, and our conversations are not empty talk; it must be something more profound. You never know when that day will come. (participant 1)

Participants were aware of their finiteness and end of the life shared with their loved ones. Participants described how they had experienced bereavement earlier in life. The loss of parents, children, partners or siblings deeply intertwined their life stories. A woman strived to come to terms with the death of her closest ones.

I still do not understand that my husband is dead, and I will not accept it. And my sisters, why did they have to die? I have no one I can talk to about this. Not in the hospital, not in the [nursing] home, not with my children. I want to go to communion, but it is challenging to kneel at the altar with my frail body. (participant 2)

She expressed loneliness and yearning for her deceased husband and siblings. The grief of losing loved ones can be brutal to alleviate when feeling abandoned. Another man alluded to the idea of living alone. He had lived a life of loneliness and refused an offer to stay in a nursing home.

I feel better at home than at the nursing home. Here I wander around by myself. I think I have come to terms with life as it is. However, it’s a little sad to live like this for years … . I would have preferred if death came quickly. Nevertheless, there is nothing I can do about it. I guess I cannot take pills that kill me either. I have never thought of that. I cannot do that. (participant 4)

He described his life as empty. His closest relatives had passed away, and his frail body could not endure the work at the farm any longer. He strived to find meaning in his life within his home’s altered and silent atmosphere, being the only one left. Thinking of ways to end his life while refusing such thoughts indicates his experience of having nothing to live for, even though taking his own life was something he could not accomplish. He had come to terms with finiteness and the existential vulnerabilities of life. Nevertheless, he enjoyed seeing other people and appreciated the weekly visits when healthcare providers delivered medicines and changed his bandage, but he said that enduring the time alone in his house between these visits was hard. These visits were the only regular human presence he experienced and were decisive in connecting him with the outside world.

Well, I think it is nice. The nurses are all very friendly, and I look forward to being visited by the home nurses. We don’t have much to talk about, but I think it is good to see them. (participant 4)

At-homeness is connected to well-being and to living a meaningful life. Participants expressed more concern about the absence of well-being than about dying. Feeling alive was especially important since frailty led to so many losses and, for some, grief and loneliness. Participants described the experience of frailty, grief and ageing that made them long for something or someone to share their experiences with. Being connected to nature, having spiritual beliefs or sharing their innermost feelings or stories seemed to enhance well-being and a sense of at-homeness.

## Discussion

This study provides new insight regarding frail older people’s experiences and understanding of at-homeness, highlighting that frailty disrupted participants’ rhythm and continuity in everyday life at home. Short-term stays at a nursing home further forced participants’ lives into a new rhythm, not in tune with their own. An overall finding was that the participants wanted to regain a feeling of at-homeness, described as an aspect of well-being despite illness (Svenaeus, 2010). The disrupted rhythm differed for participants according to their civil status and living conditions and the interdependence participants and spouses experienced in their shared home, trying to maintain the shared rhythms on which they had built their lives. The shared responsibility underscored their togetherness when attuning to a changing situation. Previous research states that the home links to self-identity and personal, societal and cultural values, beliefs, norms and meanings (Molony, 2010). Our findings show that frailty forced participants to let go of some of the things they felt were essential to maintain their experience of at-homeness.

Furthermore, our findings revealed that frailty reduced participants’ abilities. Living with a partner or significant other was crucial, and they drew strength from each other. Albeit, at the same time, dependence. The importance of relying upon someone aligns with previous research, in which frail older people left the decision-making process to a partner or close family members for the benefit of the participants and helped balance the ever-changing function and the world around them (Combes et al., 2021). However, our study further highlights that the participants wanted to continue living together in their shared home, receiving support and help from someone knowing their story. The participants’ strengths and values had changed from being a performer of practicalities to being conversation partners and tradition bearers of everyday rhythms and traditions of the home. These findings support previous research and emphasize the importance of everyday activities in familiar surroundings (Munkejord et al., 2018).

Nevertheless, participants who had lived alone in adulthood still experienced at-homeness as a commitment to their home. They described rituals and seasonal work performed in the home for generations, such as working with wood in the spring, as essential contributors to the experience of at-homeness. Earlier studies among older people support this: the home was a place that the older adults could not imagine living without, and it had become an integral part of living itself and was an intimate part of the older person’s being (Hilli & Eriksson, 2019). Participants experience a unique atmosphere pervading their home, affected by its natural surroundings, reflecting their values, beliefs, personality and way of life. The ability to follow seasonal changes throughout the year by looking out the window or opening the door is paramount, and participants do not know whether this feeling is possible to obtain when away from home. Others describe the well-being experienced while being close to nature, with its silence, peace and tranquillity leading to a feeling of at-homeness (Hemberg et al., 2020; Molony, 2010).

Our findings have similarities with previous studies describing the home as a person’s innermost space, where people have continually shaped their homes, recognized by significant others, cherished possessions and everything that mattered to them (Combes et al., 2021; Hilli & Eriksson, 2019; Molony, 2010). Participants described the home as a sanctuary they could return to, associated with familiar rhythms associated with lived life and its continuation. Once familiar and comfortable, participants perceived the home and body as less inviting and tried to adapt to their changing situation. Since participants could not maintain the house or their hygiene to their preferred standard or could not go out alone, the atmosphere in the home changed, and they may feel homeless in their own home. Hilli and Eriksson (2019) argue that one aspect of the home can be a place full of unmanageable duties, which parallels Pasveer et al. (2020), who state that the home can also be an extra burden that amplifies decay. This study emphasized that living with frailty meant that domestic duties became an overwhelming burden and participants’ description of not being at home points to a double burden since they linked at-homeness to being at home. They have lost a lot due to ageing and frailty and therefore have doubts about leaving home, trying to create and recreate the feeling of at-homeness despite illness (Søvde et al., 2022).

The introduction of home health care was a relief, and the participants appreciated help with practical tasks and health care when frailty prevented them from doing it themselves. However, this was not sufficient to regain the feeling of at-homeness, and some participants perceived it as a warning that life at home would soon be over as they knew and appreciated it. Previous research shows that introducing health care providers can threaten the home’s identity and integrity and negatively affect the participants’ way of life (Gillsjö et al., 2011). Our findings have some similar findings, but moreover, participants expressed satisfaction with the care they received at home. However, our findings show that the participants’ connection to the home, community, family and nature conflicted with their ability to perform daily activities: for instance, not being able to walk up and down stairs forced them to move to a nursing home while the house was adapted. These findings support Nilsen et al. (2021), who stated that people’s desire to live in a familiar environment sometimes conflicts with the accessibility of the housing needed for mobility.

Interestingly, participants felt safer with more people around and more professional assistance available during the temporary stays at the nursing home. Nevertheless, fixed routines and reduced opportunities to go outdoors made them feel alienated. Our findings highlight that, in addition to new routines and rhythms of the nursing home, participants felt deprived of the natural rhythms that belonged to being outdoors, such as changes in daylight, temperature and weather throughout the day, from season to season. These rhythms had previously helped them attune to nature and towards a feeling of at-homeness. These findings have similarities with previous research, showing that routines, loss of autonomy and inactivity negatively impact residents living in nursing homes (Cooney, 2012; Fæø et al., 2019; Paddock et al., 2019).

Moreover, participants found temporary stays necessary and acceptable. However, they feared moving there permanently and expressed resistance to being away from home, friends and family. They started to question their immediate future and foregrounded the idea of a permanent place in the nursing home. Previous research emphasizes that the home refers to the end as people follow their life path to find the ultimate destination for their existence. Existential issues, including their finitude, became more apparent, reinforcing a sense of limited time and moving to a nursing home mainly involved complex changes and losses that affected the individual’s well-being and identity (Dekkers, 2009). Participants described the move permanently to a nursing home as embarking on the final journey in solitude for fear of leaving everything that made their lives meaningful. They were thrown into a situation impossible to escape. Participants’ fears and opposition displayed an irreversible change of life for participants unavoidable yet experienced as an existential crisis. The strong desire to stay at home has similarities to the findings of Fæø et al. (2019) related to home-dwelling older people with dementia, describing the loss of autonomy and lack of homeliness at the nursing home followed by an aversion to moving there. Further, Fæø et al. described participants’ strong desire to stay home, although knowing that nursing home admission would be necessary at some point and that they would have to accept it. Our findings support this to a certain extent but further deepen participants’ ambivalent feelings towards moving away from home.

Participants’ insight increased as their bodies weakened, and they feared losing their homes and shelters while losing themselves from illness and aggravation. Following Svenaeus (2010), illness is linked to not being at home in the body, while Hilli and Eriksson (2019) highlighted that feeling of not belonging to a place tends to alienate and threaten a person’s existence. Nevertheless, the participants were not afraid of dying, and they described more concern about how their life would be and the urgent need to live well here and now. According to Vetlesen (2009), every human move towards their future and has ideas, hopes, and fears about it. Living with frailty was a condition that forced participants to choose what was essential in life by opting out of what was less crucial, ending up with existential conditions of human life, such as vulnerability, dependence and mortality (Heidegger, 1962). The participants experienced these conditions as marginalized, making them feel not being at home. As their current situation developed, it became clear that life was not sustainable as they lived it. Their frailty, age, illness, and disability put life itself under pressure. Our findings show that life did not always turn out as the participants hoped after returning home from a short stay at the nursing home. The practicality of the home and care services foregrounded treatment and care for health issues, leaving existential issues in the background.

Previous research underscored that frail older people often experience multidimensional losses and are frequently affected by complex and increasingly burdensome symptoms, making achieving well-being more challenging (Andrew et al., 2012; Nieboer et al., 2018). Our study underscored that being in the transition between home and nursing home and a robust and frail body represents a uniquely significant experience for frail older people, highlighting existential issues that health-care providers need to address.

## Methodological considerations

We conducted this study with frail older people in rural x. We used clear inclusion criteria with a well-known frailty model to ensure that the participants were frail. The findings have similarities with previous studies, indicating that the essential meanings highlighted might be relevant across cultural settings. Nevertheless, the cultural settings should be considered regarding the transferability of findings. Trustworthiness is essential throughout the entire research process, and this means asking how valid the knowledge is and whether it offers new knowledge about the phenomenon under study (Shenton, 2004). To ensure rigour and trustworthiness in our study, we aimed for a varied sample to increase the findings’ dependability, transferability, credibility and confirmability (Lincoln & Guba, 1985). To increase the credibility, the first author used her clinical experience when planning and preparing the interviews, preparing the interview guide, and all authors read and participated in the analysis. Variation in the sample was required to strengthen the dependability (Shenton, 2004). Consequently, we aimed to reach participants with different backgrounds. A reflexive attitude was maintained throughout the research process to safeguard confirmability. This means that we were aware of our role in interaction with the participants, empirical data, theoretical perspectives and pre-understanding that the researchers brought into the project. Regarding the transferability (Shenton, 2004), the informants were recruited from two inpatient clinics and were given care from different care contexts; this may strengthen the transferability. The limited number of participants might have limited the range of experiences described in this article. However, we consider our findings strong because we had a varied sample and rich data we have analysed to describe the essentials of a complex phenomenon. These aspects add strength to validity according to well-established criteria for validity in qualitative studies (Creswell & Creswell Báez, 2020). One strength of our study is giving voice to a vulnerable group of older people on the verge of managing at home and their perception of at-homeness, which is unique.

## Conclusion

Our study provides insight into the lived experiences with frailty related to at-homeness. Older people described the experience of frailty as an existential feeling of not being at home, emerging as inevitable reality participants have to face in their near future. The experience of being lost in transition represents a uniquely significant experience for frail older people, foregrounding existential issues and carrying the potential of at-homeness.

## Relevance for clinical practice

To unleash frail older people’s potential for at-homeness, healthcare professionals must address and meet the needs of frail older people individually. This means that planning and caring for frail older people involves more than medical decision-making and care, indicating that going beyond signs and symptoms is crucial to understanding what living with frailty is like. Understanding people’s concrete, everyday experiences is helpful in supportive care. Healthcare providers need to address existential needs and provide more holistic care than today. Our study highlighted that frail older people prefer to live well here and now instead of planning future care. However, being aware of frail older people’s potential for sudden deterioration and fluctuating health, healthcare providers should have conversations with patients about how they envisage their near future, aiming to enhance the feeling of at-homeness independent of where and how frail older people live their lives.

## Data Availability

The data is not publicly available due to privacy and ethical restrictions.

## References

[cit0001] Andrew, M. K., Fisk, J. D., & Rockwood, K. (2012). Psychological well-being in relation to frailty: A frailty identity crisis? *International Psychogeriatrics*, 24(8), 1347–11. 10.1017/S1041610212000269 22436131

[cit0002] Baldursson, S. (2002). The nature of at-homeness. *Phenomenology Online*. Retrieved April 28, 2022, from https://www.phenomenologyonline.com/sources/textorium/baldursson-stefan-the-nature-of-at-homeness

[cit0003] Brinkmann, S., & Kvale, S. (2014). *InterViews: Learning the craft of qualitative research interviewing* (3rd ed.). Sage.

[cit0004] Clegg, A., Young, J., Iliffe, S., Rikkert, M. O., & Rockwood, K. (2013). Frailty in elderly people. *The Lancet*, 381(9868), 752–762. 10.1016/S0140-6736(12)62167-9 PMC4098658 23395245

[cit0005] Combes, S., Gillett, K., Norton, C., & Nicholson, C. J . (2021). The importance of living well now and relationships: A qualitative study of the barriers and enablers to engaging frail elders with advance care planning. *Palliat Med*. Jun;35(6), 1137–1147. 10.1177/02692163211013260 PMC8189003 33934669

[cit0006] Cooney, A. (2012). ‘Finding home’: A grounded theory on how older people ‘find home’ in long‐term care settings. *International Journal of Older People Nursing*, 7(3), 188–199. 10.1111/j.1748-3743.2011.00278.x 21631889

[cit0007] Creswell, J. W., & Creswell Báez, J. (2020). *30 essential skills for the qualitative researcher* (Second ed.). Sage.

[cit0008] Dekkers, W. J. M. (2009). On the notion of home and the goals of palliative care. *Theoretical Medicine and Bioethics*, 30(5), 335–349. 10.1007/s11017-009-9121-5 19943192 PMC2785892

[cit0009] Dodge, R., Daly, A. P., Huyton, J., & Sanders, L. D. (2012). The challenge of defining well-being. *International Journal of Wellbeing*, 2(3), 222–235. 10.5502/ijw.v2.i3.4

[cit0010] Eriksson, K., & Eriksson, K. (2018). *Vårdvetenskap: Vetenskapen om vårdandet: Om det tidlösa i tiden*. Liber.

[cit0011] Fæø, S. E., Husebo, B. S., Bruvik, F. K., & Tranvåg, O. (2019). “We live as good a life as we can, in the situation we’re in” - the significance of the home as perceived by persons with dementia. *BMC Geriatrics*, 19(1), 158. 10.1186/s12877-019-1171-6 31170916 PMC6555012

[cit0012] Fried, L. P., Tangen, C. M., Waltson, J., Newman, A. B., Hirsh, C., Gottdiener, J., and McBurnie, M. A. (2001). Frailty in older adults: Evidence for a phenotype. *Journal of Gerontology: Medical Sciences*, 56(3), M146–M156. 10.1093/gerona/56.3.m146 11253156

[cit0013] Frost, R., Belk, C., Jovicic, A., Ricciardi, F., Kharicha, K., Gardner, B., Iliffe, S., Goodman, C., Manthorpe, J., Drennan, V. M., & Walters, K. (2017). Health promotion interventions for community-dwelling older people with mild or pre-frailty: A systematic review and meta-analysis. *BMC Geriatrics*, 17(1). 10.1186/s12877-017-0547-8 PMC5520298 28728570

[cit0014] Frost, R., Kharicha, K., Jovicic, A., Liljas, A. E. M., Iliffe, S., Manthorpe, J., Gardner, B., Avgerinou, C., Goodman, C., Drennan, V. M., & Walters, K. (2018). Identifying acceptable components for home‐based health promotion services for older people with mild frailty: A qualitative study. *Health & Social Care in the Community*, 26(3), 393–403. 10.1111/hsc.12526 29210136

[cit0015] Gillsjö, C., Schwartz-Barcott, D., & von Post, I. (2011). Home: The place the older adult cannot imagine living without. *BMC Geriatrics*, 11(1), 10. 10.1186/1471-2318-11-10 21410994 PMC3072327

[cit0016] Heidegger, M. (1962). *Being and time*. Basil Blackwell.

[cit0017] Hemberg, J., Nyqvist, F., Ueland, V., & Näsman, M. (2020). Experiences of longing in daily life and associations to well-being among frail older adults receiving home care: A qualitative study. *International Journal* of Qualitative *Studies* on *Health* and *Well-being*, 15(1), 1857950. 10.1080/17482631.2020.1857950 33327892 PMC7751386

[cit0018] Hilli, Y., & Eriksson, K. (2019). The home as ethos of caring: A concept determination. *Nursing Ethics*, 26(2), 425–433. 10.1177/0969733017718395 28738727

[cit0019] Hoogendijk, E. O., Afilalo, J., Ensrud, K. E., Kowal, P., Onder, G., & Fried, L. P. (2019). Frailty: Implications for clinical practice and public health. *The Lancet*, 394(10206), 1375. 10.1016/S0140-6736(19)31786-6 31609228

[cit0020] Kojima, G., Taniguchi, Y., Iliffe, S., Jivraj, S., & Walters, K. (2019). Transitions between frailty states among community-dwelling older people: A systematic review and meta-analysis. *Ageing Research Reviews*, 50, 81–88. 10.1016/j.arr.2019.01.010 30659942

[cit0021] Lekan, D. A., Collins, S. K., & Hayajneh, A. A. (2021). Definitions of frailty in qualitative research: A qualitative systematic review. *Journal of Aging Research*, 2021, 1–20. 10.1155/2021/6285058 PMC8189777 34123425

[cit0022] Lincoln, Y. S., & Guba, E. G. (1985). *Naturalistic inquiry*. Sage.

[cit0023] Merleau-Ponty, M. (2002). *Phenomenology of perception*. Taylor and Francis.

[cit0024] Ministry of Health and Care Services, Norway (2018). A full life – All your life. A quality reform for older persons. White paper no. 15 (2017–2018). Oslo: Government of Norway. https://www.helsedirektoratet.no/tema/leve-hele-livet-kvalitetsreformen-for-eldre/St%20Meld%2015%20-%20engelsk.pdf/_/attachment/inline/8561e891-57dd-447b-a184-895b739d74ce:c3c28dd80df596a4c8a05ffe6e5cf2e291198ba0/St%20Meld%2015%20-%20engelsk.pdf, accessed 30 April 2022.

[cit0025] Molony, S. L. (2010). The meaning of home: A qualitative metasynthesis. *Research in Gerontological Nursing*, 3(4), 291–307. 10.3928/19404921-20100302-02 20429493

[cit0026] Munkejord, M. C., Eggebø, H., & Schönfelder, W. (2018). Hjemme best?: En tematisk analyse av eldres fortellinger om omsorg og trygghet i eget hjem. *Tidsskrift for Omsorgsforskning*, 4(1), 16–26. 10.18261/.2387-5984-2018-01-03

[cit0027] Nieboer, A. P., Nieboer, A. P., Cramm, J. M., & Cramm, J. M. (2018). Age-friendly communities matter for older people’s well-being. *Journal of Happiness Studies*, 19(8), 2405–2420. 10.1007/s10902-017-9923-5

[cit0028] Nilsen, E. R., Hollister, B., Söderhamn, U., & Dale, B. (2021). What matters to older adults? Exploring person‐centred care during and after transitions between hospital and home. *Journal* of *Clinical Nursing*. 31, 569–581. 10.1111/jocn.15914 34117673

[cit0029] O’Neill, M., Ryan, A., Tracey, A., & Laird, L. (2020). “You’re at their mercy”: Older peoples’ experiences of moving from home to a care home: A grounded theory study. *International Journal of Older People Nursing*, 15(2), e12305–n/a. 10.1111/opn.12305 31997550

[cit0030] Öhlén, J., Ekman, I., Zingmark, K., Bolmsjö, I., & Benzein, E. (2014). Conceptual development of “at-homeness” despite illness and disease: A review. *International Journal of Qualitative Studies on Health and Well-being*, 9(1), 236770. 10.3402/qhw.v9.23677 PMC4036382 24867057

[cit0031] Österlind, J., Ternestedt, B. M., Hansebo, G., & Hellström, I. (2017). Feeling lonely in an unfamiliar place: Older people’s experiences of life close to death in a nursing home. *International Journal of Older People Nursing*, 12(1), e12129–n/a. 10.1111/opn.12129 27624362

[cit0032] Paddock, K., Brown Wilson, C., Walshe, C., & Todd, C. (2019). Care home life and identity: A qualitative case study. *Gerontologist*, 59(4), 655–664. 10.1093/geront/gny090 30085052 PMC6630159

[cit0033] Pasveer, B., Synnes, O., & Moser, I. (2020). *Ways of home making in care for later life*. Springer Singapore Pte. Limited.

[cit0034] Patton, M. Q. (2015). *Qualitative research & evaluation methods: Integrating theory and practice* (4th ed.). Sage.

[cit0035] Saarnio, L., Boström, A.-M., Hedman, R., Gustavsson, P., & Öhlén, J. (2017). Enabling at-homeness for residents living in a nursing home: Reflected experience of nursing home staff. *Journal of Aging Studies*, 43, 40–45. 10.1016/j.jaging.2017.10.001 29173513

[cit0036] Saarnio, L., Boström, A. M., Gustavsson, P., Hedman, R., & Öhlén, J. (2018). Temporally and spatially shaped meanings of at‐homeness among people 85 years and over with severe illness. *International Journal of Older People Nursing*, 13(1), e12165–n/a. 10.1111/opn.12165 28840645

[cit0037] Saarnio, L., Boström, A.-M., Hedman, R., Gustavsson, P., & Öhlén, J. (2019). Enabling at-homeness for older people with life-limiting conditions: A participant observation study from nursing homes. *Global Qualitative Nursing Research*, 6, 1–12. 10.1177/2333393619881636 PMC6806114 31673571

[cit0038] Shenton, A. K. (2004). Strategies for ensuring trustworthiness in qualitative research projects. *Education for Information*, 22(2), 63–75. 10.3233/EFI-2004-22201

[cit0039] Sjöberg, M., Edberg, A. K., Rasmussen, B. H., & Beck, I. (2019). Being acknowledged by others and bracketing negative thoughts and feelings: Frail older people’s narrations of how existential loneliness is eased. *International Journal of Older People Nursing*, 14(1), e12213–n/a. 10.1111/opn.12213 30403002

[cit0040] Søvde, B. E., Sandvoll, A. M., Natvik, E., & Drageset, J. (2022). In the borderland of the body: How home-dwelling older people experience frailty. *Scandinavian Journal of Caring Sciences*, 36(1), 255–264. 10.1111/scs.12984 33939195

[cit0041] Statistics Norway (2019). *Vesentlig mer bruk av omsorgstjenester ved passerte 85 år* [Substantially more use of care services after reaching 85 years of age]. Retrieved April 30, 2022, from https://www.ssb.no/helse/artikler-og-publikasjoner/vesentlig-mer-bruk-av-omsorgstjenester-ved-passerte-85-ar

[cit0042] Svenaeus, F. (2010). Illness as unhomelike being-in-the-world: Heidegger and the phenomenology of medicine. *Medicine, Health Care and Philosophy*, 14(3), 333–343. 10.1007/s11019-010-9301-0 21107913

[cit0043] Van Manen, M. (1990). *Researching lived experience: Human science for an action sensitive pedagogy*. State University of New York Press.

[cit0044] Van Manen, M. (2014). *Phenomenology of practice: Meaning-giving methods in phenomenological research and writing* (Vol. 13). Left Coast Press.

[cit0045] Vetlesen, A. J. (2009). *A philosophy of pain*. Reaktion Books.

[cit0046] Walker, R., Johns, J., & Halliday, D. (2015). How older people cope with frailty within the context of transition care in Australia: Implications for improving service delivery. *Health & Social Care in the Community*, 23(2), 216–224. 10.1111/hsc.12142 25427647

[cit0047] Warmoth, K., Lang, I. A., Phoenix, C., Abraham, C., Andrew, M. K., & Hubbard, R. E. (2016). Ageing and Society. 36(7), 1483. https://www.cambridge.org/core/services/aop-cambridge-core/content/view/49CD922BEDECD768F1E556019C270FEF/S0144686X1500046Xa.pdf/div-class-title-thinking-you-re-old-and-frail-a-qualitative-study-of-frailty-in-older-adults-div.pdf

[cit0048] WHO (2017). *WHO Clinical Consortium on Healthy Ageing. Report of consortium meeting 1–2 December 2016 in Geneva, Switzerland*. World Health Organization. Retrieved April 30, 2022, from https://apps.who.int/iris/bitstream/handle/10665/272437/WHO-FWC-ALC-17.2-eng.pdf

[cit0049] World Medical Association. (2013). World medical association declaration of Helsinki: Ethical principles for medical research involving human subjects. *Journal of the American Medical Association*, 310(20), 2191–2194. 10.1001/jama.2013.281053 24141714

